# Factors associated with non-response in routine use of patient reported outcome measures after elective surgery in England

**DOI:** 10.1186/1477-7525-10-34

**Published:** 2012-03-30

**Authors:** Andrew Hutchings, Jenny Neuburger, Kirstin Grosse Frie, Nick Black, Jan van der Meulen

**Affiliations:** 1 Department of Health Services Research & Policy, London School of Hygiene & Tropical Medicine, 15-17 Tavistock Place, London WC1H 9SH, UK

**Keywords:** Response bias, Mailed surveys, Patient reported outcomes, Clinical audit

## Abstract

**Background:**

Patient-reported outcome measures are increasingly being used to compare providers. We studied whether non-response rates to post-operative questionnaires are associated with patients' characteristics and organisational features of providers.

**Methods:**

131 447 patients who underwent a hip or knee replacement, hernia repair or varicose vein surgery in 2009-10 in England. Multivariable logistic regression to calculate adjusted odds ratios of non-response for characteristics of the patients and organisational characteristics of providers. Multiple imputation was used for missing patient characteristics. Providers were included as random effects.

**Results:**

Response rates to the post-operative questionnaire were 85.1% for hip replacement (n = 37 961), 85.3% for knee replacements (n = 44 422), 72.9% for hernia repair (n = 34 964), and 64.8% for varicose vein surgery (n = 14 100). Across the four procedures, there were higher levels of non-response in men (odds ratios 1.03 [95% CI 0.95-1.11] - 1.35 [1.25-1.46]), younger patients (those under 55 years 3.01 [2.72-3.32] - 6.05 [5.49-6.67]), non-white patients (1.24 [1.11-1.38] - 2.08 [1.89-2.31]), patients in the most deprived quintile of socio-economic status (1.47 [1.34-1,62] - 1.86 [1.71-2.03]), those who lived alone (1.11 [0.99-1.23] - 1.27 [1.18-1.36]) and those who had been assisted when completing their pre-operative questionnaire (1.26 [1.10-1.46] -1.67 [1.56-1.79]). Non-response rates were also higher in patients who had poorer pre-operative health (three or more comorbidities: 1.14 [0.96-1.35] - 1.45 [1.30-1.63]). Providers' patient recruitment rates before surgery and the timing of pre-operative questionnaire administration did not affect the rates of response to post-operative questionnaires.

**Conclusion:**

If non-response can be shown to be associated with outcome, then rates of non-response to post-operative questionnaires would need to be taken into account when these measures are being used to compare the performance of providers or to evaluate surgical procedures.

## Background

In April 2009 the National Health Service (NHS) in England embarked on a national programme to collect pre- and post-operative patient reported outcome measures (PROMs) in all patients undergoing hip replacement, knee replacement, groin hernia repair, and varicose vein (VV) surgery [1]. Participation in the programme is mandatory for all National Health Service (NHS) and independent sector providers (hospitals and treatment centres) of NHS-funded care, although individual patients can choose not to take part. Potential uses of the data include comparisons of the performance of providers, audit of the equity of care, evaluation of the cost-effectiveness of different procedures, and estimation of the amount of health gain for populations. There are plans to extend the programme to additional surgical procedures. The use of PROMs in long-term conditions is being explored. The only previous use of PROMs on a national scale is for hip surgery in Sweden [2].

Pilot studies for the PROMs Programme suggested that response rates to mailed post-operative questionnaires of between 75% and 90% were achievable, dependent on the operation performed [3,4]. Whilst these are high, rates vary between health care providers from 30% to 100% [5] which might give rise to biased comparisons if systematic differences exist between responders and non-responders.

Although there is an extensive literature on factors influencing response in household and longitudinal surveys, [6-8] there is less evidence on surveys of hospital patients. In England, the response to the NHS inpatient survey tends to be lower amongst men, younger patients and non-white patients [9]. However, the evidence is more mixed for surveys of specific patient populations: some studies have reported older patients are less likely to respond [10,11]; some have found the opposite [12] and others report either no association with age [9] or those at both extremes (youngest and oldest) are less likely to respond [13]. As regards the sex of patients, several studies have found no association [11-14]. Most studies have found that patients in worse health at the time of their hospital admission are less likely to respond to later questionnaires [10,11,15,16] although one study did not find such an association [17]. The earlier small study we conducted of PROMs in surgery found that response was higher among older and less deprived patients [4].

The importance of responder bias is evident in the many studies that have reported that non-responders' have poorer outcomes [10,11,13-20]. Non-response therefore carries the risk of over-estimating the outcomes of providers with lower response rates. The aim of this study was to establish whether non-response is associated with patients' socio-demographic and clinical characteristics or with organisational factors for four surgical procedures. It was observational rather than testing a hypothesis.

## Methods

### Data sources

The analyses are based on data collected on patients who underwent surgery during the first year of the programme (1 April 2009 to 31 March 2010). Patients were invited to complete a pre-operative questionnaire either when attending the pre-operative assessment clinic (which might be several weeks before their operation) or on admission on the day of surgery. The completed questionnaires were sent by the provider to the NHS Information Centre (IC) for processing. Providers were made up of NHS hospitals, treatment centres (ambulatory surgical centres) and independent (private) hospitals.

The NHS IC links data from patients' pre-operative questionnaires to data on their episode of care recorded in Hospital Episode Statistics (HES) to identify each patient's date of surgery. Post-operative questionnaires are then mailed to patients' homes, from a national source, three (hernia repair and VV surgery) or six (hip and knee replacements) months following surgery.

Linkage to HES was successful for 75% of patients. For those patients whose pre-operative questionnaire could not be linked to HES and for whom, therefore, the date of surgery was unknown, the post-operative questionnaires were sent out six (hernia repair and VV surgery) and nine (hip and knee replacements) months after the date of completion of the pre-operative questionnaire.

Patients were excluded from the analysis if their operation was cancelled or they died before the post-operative questionnaire was due to be sent. Patients who had not returned a completed post-operative questionnaire by the end of August 2011 were classified as non-responders.

### Explanatory variables

Pre-operative questionnaires obtained data on the socio-demographic characteristics of patients comprising age, sex, living arrangements (alone or otherwise) and if the patient required assistance completing the pre-operative questionnaire. Data on ethnicity and socio-economic deprivation were not included and had to be obtained from the linked HES record. For patients without a linked HES record, or with missing information in the linked record, we searched for data on ethnicity and post code (from which socio-economic status was derived) in HES records of previous hospital admissions. Deprivation was measured using the Index of Multiple Deprivation (IMD) [21] and was categorised in five groups based on quintiles derived from the national distribution.

Data on several aspects of patients' clinical status were obtained from their pre-operative questionnaires: duration of symptoms; previous surgery for the same condition; comorbidities [4]; the EuroQol EQ-5D index score [22]; and for three procedures, a condition-specific PROM - Oxford Hip Score, [23] Oxford Knee Score, [24] and Aberdeen Varicose Vein Questionnaire [25].

Organisational factors comprised the type of provider (NHS or independent sector), when the pre-operative questionnaire was administered (pre-operative assessment clinic or day of admission), and the patient recruitment rate to the PROMs Programme. Data on questionnaire administration was missing for patients without a linked HES record because the date of surgery was derived from HES. Data on provider recruitment rates (proportion of eligible patients recruited to the survey), available from the NHS IC, [5] was calculated as the number of completed pre-operative questionnaires divided by the number of eligible patients, according to HES, for each procedure. Recruitment rates were not estimated for providers with fewer than 50 eligible patients.

Multiple imputation by chained equations was used to impute values for all missing pre-operative variables (except for organisational factors) for multivariable analysis under the assumption that data were missing at random [26,27]. Ten data sets were imputed using the ICE command in Stata. Missing data were imputed from the pre-operative variables, provider level mean deprivation, provider level proportion reporting non-white ethnicity, and response to the post -operative questionnaire.

### Data analysis

For each operation, the response rate to the post-operative questionnaire was estimated by each patient characteristic and organisational factor. Multivariable logistic regression models were used to estimate odds ratios of non-response with 95% confidence intervals adjusted for all patient characteristics and organisational factors, except provider recruitment rate. Estimates for timing of pre-operative questionnaire administration were based on an analysis of those patients for whom date of surgery was available through linkage with HES. The impact of differences in provider recruitment on non-response was examined separately in a logistic regression analysis, adjusted for other characteristics, with provider included in the model as a random effect to allow for the non-independence of observations within providers. No attempt was made to impute outcomes for non-responders.

## Results

### Sample size and response rates

Between 1 April 2009 and 31 May 2010, 135 474 patients completed a pre-operative questionnaire. After excluding patients who had died (927) or whose surgery had been cancelled (3100), there were 131 447 patients for analysis: 37 961 hip replacements, 44 422 knee replacements, 34 964 hernia repair, and 14 100 VV surgery. Response rates were higher for hip (85.1%) and knee replacements (85.3%) than for hernia repair (72.9%) and VV surgery (64.8%).

### Patients' socio-demographic characteristics

Most hernia surgery patients were men (89%) whereas women formed the majority of patients having the other three procedures (Table 1). Patients having hip and knee replacements were, on average, older than patients having hernia and VV surgery (mean ages 67.7 and 68.7 versus 57.4 and 49.7 years, respectively.) The majority of patients were white. The percentage of non-white patients was largest (6.2%) for VV surgery and smallest (1.8%) for hip replacements. For all procedures, except VV surgery, patients living in the most deprived areas were under-represented given that around 20% of the patients would be expected in each national IMD quintile. Around a quarter of orthopaedic patients lived alone compared with 15% of hernia and VV surgery patients. About a fifth of orthopaedic patients had assistance in completing their pre-operative questionnaire compared with 12% for groin hernia and 8% for VV surgery.

**Table 1:** Patients' socio-demographic characteristics and response by procedure

		Hip replacement		Knee replacement		Hernia repair		VV surgery	
		n (%)	Responded%	n (%)	Responded%	n (%)	Responded%	n (%)	Responded%
**Sex**	Female	22,123 (58.7)	85.4	24,631 (55.8)	85.7	3,712 (10.7)	69.7	8,890 (63.3)	66.5
	Male	15,592 (41.3)	84.7	19,527 (44.2)	85.1	31,117 (89.3)	73.3	5,153 (36.7)	62.1
	missing	246		264		135		57	
**Age (years)**	under 55	4,643 (12.3)	71.7	3,481 (7.8)	71.7	13,814 (39.6)	55.0	8,762 (62.2)	56.2
	55-64	8,443 (22.3)	84.5	10,938 (24.7)	83.7	8,141 (23.3)	80.8	2,914 (20.7)	77.2
	65-74	13,595 (35.9)	88.8	16,847 (38.0)	87.8	7,524 (21.6)	87.6	1,736 (12.3)	81.5
	75 and over	11,198 (29.6)	86.6	13,058 (29.5)	87.2	5,408 (15.5)	86.8	668 (4.7)	81.4
	missing	82		98		77		20	
**Ethnicity**	White	33,843 (98.2)	85.6	38,877 (94.7)	86.6	28,422 (94.4)	74.7	11,757 (93.8)	66.4
	Non-white	636 (1.8)	68.2	2,169 (5.3)	68.6	1,675 (5.6)	57.1	771 (6.2)	51.0
	missing	3,482		3,376		4867		1,572	
**IMD quintile**	Most deprived	4,280 (11.9)	77.5	6,116 (14.5)	79.5	5,243 (15.9)	61.3	2,832 (20.9)	55.9
	2nd	6,268 (17.5)	82.4	8,179 (19.3)	83.6	6,065 (18.4)	68.0	2,847 (21.1)	63.6
	3 rd	8,141 (22.7)	86.0	9,539 (22.5)	86.1	7,141 (21.7)	75.4	2,901 (21.4)	67.3
	4th	8,901 (24.8)	87.6	9,691 (22.9)	87.9	7,252 (22.0)	78.4	2,749 (20.3)	70.9
	Least deprived	8,278 (23.1)	88.5	8,779 (20.8)	89.1	7,271 (22.0)	80.7	2,198 (16.3)	71.0
	missing	2,093		2,118		1,992		573	
**Living**	Living with others	26,583 (72.4)	85.8	32,170 (75.2)	85.8	28,611 (84.2)	73.1	11,716 (85.4)	64.8
**arrangements**	Living alone	10,119 (27.6)	83.8	10,613 (24.8)	84.9	5,376 (15.8)	72.7	2,002 (14.6)	66.4
	missing	1,259		1,639		977		382	
**Assisted with**	No	29,731 (78.4)	86.4	34,680 (79.1)	87.2	30,622 (88.2)	73.4	12,907 (92.3)	65.2
**questionnaire**	Yes	8,108 (21.6)	80.5	9,144 (20.9)	78.4	4,103 (11.8)	69.8	1,071 (7.7)	60.5
**completion**	missing	482		598		239		122	

### Patients' clinical characteristics

Patients undergoing VV surgery were more likely to have undergone previous surgery for the same condition (39%) than patients undergoing the other three procedures (8-13%) (Table 2). The duration of symptoms also varied by procedure: 63.3% of hernia surgery patients had symptoms for less than one year compared with 73.4% of VV surgery patients having had symptoms for more than five years. Patients undergoing a joint replacement had more comorbidities, which corresponds to their older age. This was also reflected in the distribution of health-related quality of life (QoL) scores, measured by the EQ-5D. Mean EQ-5D scores were 0.34 for hip and 0.40 for knee replacements, 0.79 for hernia repair, and 0.76 for VV surgery.

**Table 2:** Patients' clinical characteristics and response by procedure

		Hip replacement		Knee replacement		Hernia repair		VV surgery	
		n (%)	Responded%	n (%)	Responded%	n (%)	Responded%	n (%)	Responded%
**Previous surgery**	No	33,738 (89.7)	85.7	40,468 (91.9)	85.9	30,318 (87.3)	73.0	8,530 (61.0)	63.4
	Yes	3,882 (10.3)	80.2	3,560 (8.1)	79.8	4,399 (12.7)	73.2	5,459 (39.0)	67.0
	missing	341		394		247		111	
**Duration of**	0-5 years	30,039 (79.9)	86.0	25,138 (57.1)	86.2	21,960 (63.3)	75.0	3,702 (26.6)	59.0
symptoms*	> 5 years	7,549 (20.1)	81.7	18,916 (42.9)	84.2	12,722 (36.7)	69.5	10,234 (73.4)	67.0
	missing	373		368		282		164	
**Comorbidities**	none	16,125 (42.5)	85.9	16,058 (36.3)	86.2	18,066 (51.7)	67.6	7,925 (56.2)	62.6
	1	13,556 (35.7)	86.0	16,574 (37.3)	86.1	9,983 (28.5)	78.1	3,756 (26.6)	66.3
	2	5,804 (15.3)	83.3	8,040 (18.1)	84.6	4,337 (12.4)	80.9	1,445 (10.3)	70.9
	3 or more	2,476 (6.5)	78.8	3,750 (8.4)	79.7	2,578 (7.4)	76.8	974 (6.9)	68.1
	missing	0		0		0		0	
**Procedure specific**	Worst	14,122 (37.5)	80.5	16,617 (37.7)	80.0	-	-	4,621 (33.3)	62.6
**health status by**	Middle	10,982 (29.2)	86.5	13,706 (31.1)	87.4	-	-	4,622 (33.3)	65.4
**tertile**	Best	12,541 (33.3)	89.2	13,725 (31.2)	89.7	-	-	4,622 (33.3)	66.8
	missing	316		374		-	-	235	
**EQ5D index by**	Worst QoL	13,062 (36.3)	80.7	16,498 (39.2)	81.3	12,957 (38.3)	69.1	4,620 (34.4)	60.1
**tertile**	Middle	11,559 (32.1)	86.4	21,514 (51.1)	87.9	9,894 (29.3)	75.3	5,503 (40.9)	66.1
	Best QoL	11,361 (31.6)	89.5	4,059 (9.7)	89.8	10,960 (32.4)	75.6	3,319 (24.7)	70.4
	missing	1,979		2,351		1,153		658	

*up to 1 year and > 1 year for groin hernia; QoL = quality of life

### Organisational factors

Completion of the pre-operative questionnaire on day of admission was more common for hernia and VV surgery patients (Table 3). For VV surgery, this reflects the lack of need for pre-operative assessment as many will not be undergoing a general anaesthetic. Around 16% to 20% of orthopaedic procedures and hernia repair were carried out by independent providers, whilst this was true for only 8% of VV procedures. Rates of recruitment to the PROMs Programme of 70% or more were commoner for hip and knee replacement (almost 60% of providers) than for the other procedures (20-30% of providers).

**Table 3:** Organisational factors and patient response by procedure

		Hip replacement		Knee replacement		Hernia repair		VV surgery	
		n (%)	Responded%	n (%)	Responded%	n (%)	Responded%	n (%)	Responded%
**Administration of**	On admission	4,671 (16.5)	85.7	5,526 (18.3)	86.5	9,233 (37.4)	75.8	5,996 (53.1)	67.2
questionnaire*	Before admission	23,650 (83.5)	87.2	24,664 (81.7)	88.2	15,428 (62.6)	78.1	5,289 (46.9)	68.5
		1,418		1,883		586		439	
**Provider type**	NHS	30,514 (80.4)	84.3	36,228 (81.6)	84.5	29,265 (83.7)	73.0	12,951 (91.9)	64.8
	Independent	7,477 (19.6)	88.2	8,191 (18.4)	88.8	5,699 (16.3)	72.8	1,149 (8.1)	65.4
	missing	0		3		0		0	
**Provider**	below 70%	13,721 (42.0)	84.6	15,923 (40.8)	85.1	22,449 (73.2)	72.8	9,838 (69.8)	63.7
recruitment rate**	70% or more	18,962 (58.0)	84.7	23,151 (59.2)	84.8	8,226 (26.8)	73.3	2,910 (20.6)	67.5
	missing	5,278		5,348		4,289		1,352	

* restricted to questionnaires linked to Hospital Episode Statistics episodes

** restricted to providers with a minimum of 50 eligible episodes

### Association between patient/organisational characteristics and non-response

The adjusted odds ratios of the pre-operative patient characteristics and organisational factors for non-response to a post-operative questionnaire are shown in Table 4. Odds ratios of patients' socio-demographic characteristics show consistent patterns across the four procedures. Non-response rates were higher in men (odds ratio 1.03 [95% CI 0.95-21.11] - 1.35 [1.25-1.46]), those under 55 years (3.01 [2.72-3.32] - 6.05 [5.49-6.67]), non-white patients (1.24 [1.11-1.38] - 2.08 [1.89-2.31]), those in the most deprived IMD quintile (1.47 [1.34-1.62] - 1.86 [1.71-2.03]), those who lived alone (1.11 [0.99-1.23] - 1.27 [1.18-1.36]), and those who had assistance in completing the pre-operative questionnaire (1.26 [1.10-1.46] - 1.67 [1.56-1.79]).

**Table 4:** Adjusted odds ratios for non-response by procedure

	Adjusted odds ratio (95% confidence interval)			
	Hip replacement	Knee replacement	Hernia repair	VV surgery
**Socio-demographic characteristics**				
Sex				
Female	1.00	1.00	1.00	1.00
Male	1.12 (1.05-1.19)	1.15 (1.09-1.22)	1.03 (0.95-1.11)	1.35 (1.25-1.46)
Age category (years)				
Under 55	3.06 (2.78-3.37)	3.01 (2.72-3.32)	6.05 (5.49-6.67)	4.08 (3.29-5.06)
55-64	1.48 (1.35-1.61)	1.50 (1.38-1.62)	1.81 (1.63-2.00)	1.50 (1.21-1.88)
65-74	0.97 (0.90-1.05)	1.05 (0.98-1.13)	1.05 (0.95-1.17)	1.13 (0.89-1.42)
75 and over	1.00	1.00	1.00	1.00
Ethnicity				
White	1.00	1.00	1.00	1.00
Non-white	1.58 (1.32-1.88)	2.08 (1.89-2.31)	1.24 (1.11-1.38)	1.25 (1.08-1.47)
IMD quintile				
Most deprived	1.55 (1.40-1.72)	1.47 (1.34-1.62)	1.86 (1.71-2.03)	1.49 (1.31-1.69)
2nd	1.34 (1.21-1.47)	1.29 (1.17-1.41)	1.60 (1.47-1.73)	1.21 (1.07-1.38)
3 rd	1.12 (1.02-1.23)	1.18 (1.07-1.29)	1.25 (1.15-1.36)	1.13 (1.00-1.28)
4th	1.04 (0.95-1.14)	1.06 (0.97-1.17)	1.13 (1.04-1.23)	0.98 (0.87-1.12)
Least deprived	1.00	1.00	1.00	1.00
Living arrangements				
Not living alone	1.00	1.00	1.00	1.00
Living alone	1.27 (1.18-1.36)	1.21 (1.13-1.29)	1.16 (1.08-1.25)	1.11 (0.99-1.23)
Assisted in completing questionnaire				
Not assisted	1.00	1.00	1.00	1.00
Assisted	1.49 (1.39-1.60)	1.67 (1.56-1.79)	1.53 (1.41-1.66)	1.26 (1.10-1.46)
**Clinical characteristics** Previous surgery				
No	1.00	1.00	1.00	1.00
Yes	1.42 (1.30-1.55)	1.36 (1.25-1.49)	1.14 (1.05-1.23)	1.05 (0.97-1.14)
Duration of symptoms*				
Up to 5 years	1.00	1.00	1.00	1.00
> 5 years	1.13 (1.05-1.21)	1.03 (0.98-1.09)	1.26 (1.19-1.32)	0.84 (0.78-0.92)
Comorbidities				
None	1.00	1.00	1.00	1.00
1	1.06 (0.99-1.13)	1.00 (0.94-1.07)	0.91 (0.85-0.97)	1.01 (0.93-1.10)
2	1.25 (1.15-1.37)	1.04 (0.96-1.12)	0.95 (0.86-1.04)	0.99 (0.86-1.14)
3 or more	1.45 (1.30-1.63)	1.24 (1.12-1.37)	1.19 (1.06-1.34)	1.14 (0.96-1.35)
Condition-specific PROM				
per 10 point worse score	1.17 (1.11-1.24)	1.15 (1.09-1.21)	-	1.06 (1.02-1.11)
EQ-5D index score				
per 0.1 point worse score	1.04 (1.03-1.06)	1.05 (1.04-1.06)	1.06 (1.05-1.08)	1.07 (1.05-1.09)
**Organisational factors**				
Administration of pre-operative questionnaire**				
On admission	1.06 (0.96-1.16)	1.06 (0.97-1.16)	1.03 (0.96-1.10)	1.11 (1.02-1.21)
Before admission	1.00	1.00	1.00	1.00
Provider type				
NHS	1.14 (1.06-1.24)	1.24 (1.15-1.34)	1.07 (1.00-1.15)	1.08 (0.94-1.23)
Independent	1.00	1.00	1.00	1.00
Provider recruitment***				
per 20% point increase	1.02 (0.98-1.07)	1.03 (0.99-1.06)	1.02 (0.98-1.07)	0.99 (0.94-1.04)

* up to and more than 1 year for hernia repair

** linked records only

*** restricted to providers with at least 50 eligible patients

Adjusted odds ratios of the clinical characteristics demonstrate that non-response was higher among patients who had previously undergone surgery for their condition (odds ratio 1.05 [0.97-1.14] - 1.42 [1.30-1.55]). Duration of symptoms was less consistent in its impact on non-response. A longer duration was associated with non-response in hip replacement and hernia repair but for VV surgery a shorter duration increased non-response. There was no significant association between symptom duration and non-response for knee replacement. There was a pattern of patients with worse health (comorbidity, EQ-5D, and condition-specific PROMs) having higher levels of non-response.

There was no evidence that the time of administration of the pre-operative questionnaire was associated with post-operative non-response except for VV surgery patients who had a slightly higher non-response rate if the pre-operative questionnaire had been administered on admission (OR 1.11 [95% CI 1.02-1.21]). For orthopaedic surgery there was evidence of a higher non-response rate in patients treated by NHS than independent providers (1.08 [0.94-1.23] - 1.24 [1.15-1.34]). According to the adjusted odds ratio of the providers' recruitment rate (Table 4), there is no evidence that providers who are successful in recruiting a high proportion of patients will have higher levels of non-response because they recruit more 'reluctant participants'. The odds ratio of a 20% increase in recruitment rate only ranged from 0.99 [0.94-1.04] to 1.03 [0.99-1.06] for the four procedures.

## Discussion

### Main findings

Patients who did not respond to the post-operative questionnaire tended to have more severe primary conditions and a poorer pre-operative quality of life. In addition, response was lower in patients who were men, younger, non-white, deprived, living alone, and having undergone previous similar surgery. Orthopaedic patients were less likely to respond if they had been treated in an NHS rather than independent provider. The provider's pre-operative recruitment rate and the timing of pre-operative questionnaire administration were not associated with the post-operative response rate.

### Comparison with previous research on non-response to post-operative questionnaires

We have studied many more determinants of the response rate to PROM questionnaires mailed to patients after a hospital episode than have been considered previously in published studies. Only the impact of age, sex and health status have been studied before (apart from in our own small study [4] in which we found more deprived people were less likely to respond). Our finding in this study that younger patients (under 55 years in particular) are less likely to respond is consistent with our previous study [4] and a study in Norway of spinal surgery patients, [12] whereas two other studies have found the opposite [10,11]. The reason for this lack of consistency is unclear. It may reflect the influence of the medical condition and the clinical treatment: studies finding older people less likely to respond were based on investigations of men undergoing prostate surgery [10] and patients with acute coronary syndrome [11].

Our finding that men were less likely to respond is at odds with the literature, which has reported no association with sex for knee replacement [14] or for a mixed population of hospital patients [13]. Again, the reason for this difference in observed association is unclear.

In contrast, the association we observed of non-response with poorer health has been consistently reported in a wide variety of patients: prostate surgery [10], acute coronary syndrome [11], shoulder surgery [15], all hospital admissions [16], and our previous study of elective surgery [4].

### Limitations of the study

The first limitation is that date of surgery was not available for 25% of the patients because their pre-operative questionnaire could not be linked to their HES record. As a result, these patients were not included in the analysis considering the timing of administration of pre-operative questionnaires. It is reassuring that in those patients whose PROMs could be linked to HES, we did not find evidence that the timing of the administration of the pre-operative questionnaires influenced response rates.

Second, it is important to take into account the volume of the data and the fact that they are observational. The large volume allows effects to be estimated with great precision, i.e. narrow confidence intervals, so even small effects are regarded as highly statistically significant. However, there is scope for selection biases and residual confounding such that even modest confounding could alter the apparent significance of a small effect.

### Implications of findings

The implications of non-response for the use of PROMs data will depend on the extent to which non-response is associated with outcomes. Previous research has shown that those who do not respond have worse outcomes [10,11,13-20]. If this were to prove true for elective surgery and non-response is not taken into account, outcomes will be over-estimated, particularly among providers with high non-response rates.

A number of approaches have been suggested to adjust for bias resulting from non-response [2,3,11,26]. Both weighting and multiple imputation are options for including the outcomes of patients who do not respond, based on the outcomes of patients for whom complete data are available [26,27]. If this approach is adopted, this study has identified the patient and organisational factors that should be included in such models. However, the validity of these techniques is dependent on the assumption that the probability that data are missing does not depend on the value of the missing item having adjusted for observed characteristics (ie those that have been measured). This, however, may be problematic for PROMs data if non-response bias associated with the outcome is not predicted by observed characteristics (ie characteristics for which measurements do not exist). In such circumstances, index function models, including the Heckman method, would need to be employed [10,11,13-20].

## Conclusions

If non-response can be shown to be associated with outcome using PROMs in elective surgery, then rates of non-response to post-operative questionnaires would need to be taken into account when these measures are being used to compare the performance of providers or to evaluate surgical procedures.

## Competing interests

The authors declare that they have no competing interests.

## Authors' contributions

AH and JvdM conceived and designed the analyses; AH, JN and KGF carried out the analyses; AH and NB led the drafting which all authors contributed to. All authors read and approved the final manuscript
